# C2RL: Convolutional-Contrastive Learning for Reinforcement Learning Based on Self-Pretraining for Strong Augmentation

**DOI:** 10.3390/s23104946

**Published:** 2023-05-21

**Authors:** Sanghoon Park, Jihun Kim, Han-You Jeong, Tae-Kyoung Kim, Jinwoo Yoo

**Affiliations:** 1 Graduate School of Automotive Engineering, Kookmin University, Seoul 02707, Republic of Korea; ppp7326@naver.com (S.P.); wkdrns3847@gmail.com (J.K.); 2 Department of Electrical Engineering, Pusan National University, Busan 46241, Republic of Korea; hyjeong@pusan.ac.kr; 3 Department of Electronic Engineering, Gachon University, Seongnam 13120, Republic of Korea; tkkim@gachon.ac.kr; 4 Department of Automobile and IT Convergence, Kookmin University, Seoul 02707, Republic of Korea

**Keywords:** deep reinforcement learning, self-supervised learning, contrastive learning, generalization, data augmentation, network randomization

## Abstract

Reinforcement learning agents that have not been seen during training must be robust in test environments. However, the generalization problem is challenging to solve in reinforcement learning using high-dimensional images as the input. The addition of a self-supervised learning framework with data augmentation in the reinforcement learning architecture can promote generalization to a certain extent. However, excessively large changes in the input images may disturb reinforcement learning. Therefore, we propose a contrastive learning method that can help manage the trade-off relationship between the performance of reinforcement learning and auxiliary tasks against the data augmentation strength. In this framework, strong augmentation does not disturb reinforcement learning and instead maximizes the auxiliary effect for generalization. Results of experiments on the DeepMind Control suite demonstrate that the proposed method effectively uses strong data augmentation and achieves a higher generalization than the existing methods.

## 1. Introduction

**Figure 1:**
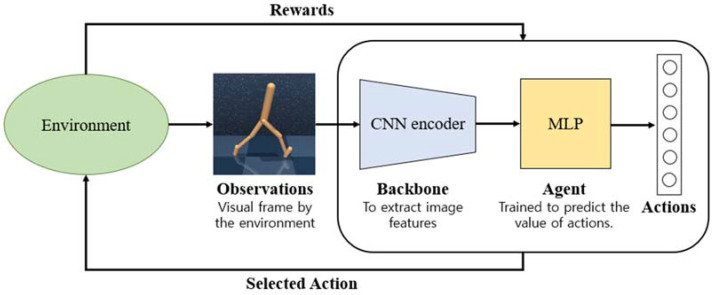
Vision-based reinforcement learning architecture.

Since the advent of AlphaGo, the potential of deep reinforcement learning has been demonstrated, and it has been applied in various fields, such as autonomous driving and automated robots. As Figure 1 shows, the combination of reinforcement learning and deep neural networks allows control tasks to be performed using high-dimensional observations, such as, images [1]. Notable successes include learning to play various games from raw images (board games [2] and video games [3,4]), controlling a car from a camera frame in the virtual environment [5], solving complicated problems from camera observations [6,7,8], and picking up objects in the real world [9].

However, the use of high dimensional observations, such as raw images, may lead to sample inefficiency [10,11]. In other words, learning the same number of steps shows a lower performance when using images rather than using a low-dimensional state vector. Among many studies, CURL increases the sample efficiency by learning the similarity between the input frames through contrastive learning, which is a self-supervised learning method that learns to extract richer representation from images while contrasting the query and key [12]. However, due to overfitting in the training environment, the reinforcement learning performance deteriorates even with minor background changes in the test environment that do not affect the action selection. In other words, in the unseen environment that is semantically similar to the seen environment, the improvement in the sample efficiency through contrastive learning is not guaranteed, and this is called a generalization problem in vision-based deep reinforcement learning [13,14].

Input image data are typically augmented to ensure a robust performance even in environments that the model has not observed [15]. Learning from various input distributions through augmentation can help prevent over-fitting in the training environment. In addition, data augmentation is essentially used for contrastive learning. Stronger data augmentation results in more effective contrastive learning, the auxiliary task of reinforcement learning, and generalization. However, the use of strong augmentation is limited because a large change in the input frame disturbs the downstream task (here, via reinforcement learning) [16]. By preventing the adverse effect of strong augmentation on reinforcement learning, the benefits of contrastive learning can be maximized, and generalization performance can be enhanced.

To improve the generalization of vision-based reinforcement learning, we propose a convolutional–contrastive learning for reinforcement learning (C2RL): a simple architecture that can be added to most reinforcement learning frameworks. Furthermore, we propose a self-pretraining method to overcome the trade-off associated with the augmentation strength and use strong augmentation for both reinforcement learning and contrastive learning without performance degradation. (i) Until the initial steps of the training stage, reinforcement learning and contrastive learning are performed without strong augmentation, such as random convolution. (ii) After training the encoder through self-pretraining, strong data augmentation, such as random convolution, is applied to the input frame and reinforcement, and contrastive learning is continued for the remaining training period. (iii) Although the input data significantly change due to strong augmentation (random convolution), robust feature extraction is possible, which does not significantly degrade the performance of reinforcement learning. (iv) Contrastive learning can induce a greater auxiliary effect on reinforcement learning due to strong augmentation.

**Figure 2:**
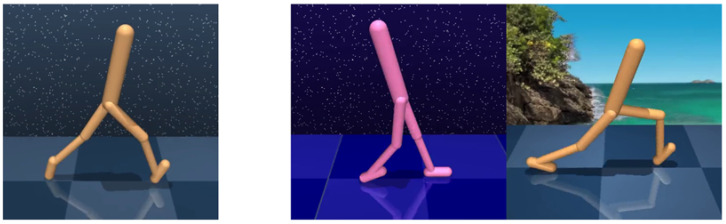
**Left:** Training environment (seen environment) of DMControl. **Right:** Test environments (unseen environments) of DMControl generalization benchmark (Color-hard and Video-easy mode) [17].

One of the greatest contributions of this study is that strong augmentation is used more effectively in our method than when the same strong augmentation is applied consistently throughout training. Furthermore, our study introduces a new attempt on how to efficiently use image data in reinforcement learning. None of the existing studies have focused on contrastive learning using random convolution, despite its potential in achieving a stronger auxiliary effect. Experiments are performed in two modes of the DeepMind Control (DMControl) suite, as shown in Figure 2. The proposed approach significantly outperforms the existing generalization methods in both statically and dynamically changing test environments.

## 2. Related Work

### 2.1. Soft Actor Critic (SAC)

For continuous control from raw images, we use the SAC, which is a state-of-the-art, off-policy reinforcement learning algorithm that maximizes the expected sum of rewards [18]. The agent outputs action $a_{t}$ from frame observations $o_{t}$, which are stored as transitions in the replay buffer D with reward $r_{t}$. The parameters of the SAC are *ψ* of the state value function $V_{\psi \;}$, *θ* of the soft Q-function $Q_{\theta }$, and *ϕ* of policy $\pi_{\phi }$. To learn a critic $Q_{\theta }$, the critic parameters are trained by minimizing the Bellman error using transitions sampled from replay buffer D:

(1) $$J_{Q_{\theta }}=E_{\left(o_{t}\;,a_{t}\right)\sim D}\;{\left[\left(Q_{\theta }\left(o_{t},\;a_{t}\right)-\left(r_{t}+\gamma V_{\psi \;}(o_{t+1}\right)\right)\right)}^{2}]$$

The state value is estimated by sampling an action from the current policy $\pi_{\phi }$, and ${\bar{Q}}_{\theta }$ denotes an exponential moving average of the critic network:

(2) $$V_{\psi \;}(o_{t+1})=E_{a\prime \sim \pi_{\phi }}\;[({\bar{Q}}_{\theta }\left(o_{t+1},\;a\prime \right)-\alpha \;log\pi_{\phi }(a\prime |\;o_{t+1})]$$

The policy parameter *ϕ* is trained by minimizing the divergence from the exponential of the soft-Q function, and α is a temperature parameter for the stochasticity of the optimal policy:

(3) $$J_{\pi_{\phi }}=-E_{a_{t}\sim \pi_{\phi }}\;[(Q_{\theta }\left(o_{t},\;a_{t}\right)-\alpha \;log\pi_{\phi }(a_{t}|o_{t})]$$

### 2.2. Self-Supervised Learning

Self-supervised learning, an unsupervised learning strategy, is aimed at learning pretext tasks to improve the downstream task performance [19,20]. The trained model can extract rich representations from unlabeled data by learning appropriate pretext tasks that can facilitate downstream tasks, such as classification, object detection, or reinforcement learning, and can utilize them through transfer learning [21]. Recently, self-supervised learning models, such as MoCo [22], SimCLR [23], BYOL [24], and BERT [25], have made great advancements in natural language processing and computer vision tasks, and have also been actively applied to vision-based reinforcement learning.

**Figure 3:**
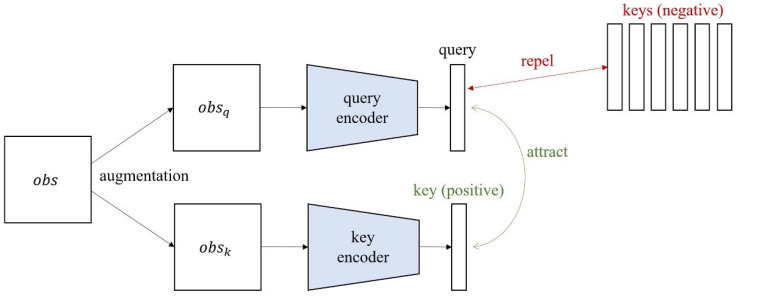
Conventional contrastive learning architecture.

Self-supervised learning can be divided into several types according to the pretext task. Among them, contrastive learning is a self-supervised learning method aimed at increasing the similarity between positive image pairs and decreasing the similarity between negative image pairs [26]. As shown in Figure 3, to define the positive and negative pairs, the input image is randomly augmented twice with each image acting as the query and key image. Based on the query, the key augmented from the same image is defined as the positive pair, and keys augmented from other images are defined as negative pairs. Contrastive learning allows a query encoder to extract rich representation vectors from unlabeled images, thereby improving the performance of downstream tasks such as reinforcement learning. In our study, InfoNCE is used as the loss function for contrastive learning. In Equation (4), *q* is the query for contrast; $k_{+}$ and $k_{i}$ are the positive and negative keys, respectively; and W is a matrix for bilinear products [27]. Through the log loss of a K-way softmax classifier with label $k_{+}$, the encoder can learn embeddings to determine the similarity between the query and keys.

(4) $$L_{NCE}=log\frac{exp\left(q^{T}Wk_{+}\right)}{exp\left(q^{T}Wk_{+}\right)+\sum_{i=0}^{K-1}exp\left(q^{T}Wk_{i}\right)\;}$$

### 2.3. Network Randomization

Random networks have been used to improve the various performance metrics associated with deep reinforcement learning. For example, researchers focusing on ensemble-based approaches used random networks to improve the uncertainty estimation and exploration of deep reinforcement learning [28]. Moreover, in unexplored state recognition tasks, randomly initialized neural networks were used to define intrinsic rewards for unexplored state visits [29]. In this study, we use a random network for improving the generalization in vision-based reinforcement learning. The input image is randomized by a single layer CNN with a kernel size of 3. Additionally, its output is padded in order to be in the same dimension as the input. For every training iteration, parameter ω is reinitialized with a prior distribution, such as Xavier normal distribution [30].

(5) $$obs_{conv}=f_{\omega }\left(obs_{origin}\right)$$

**Figure 4:**
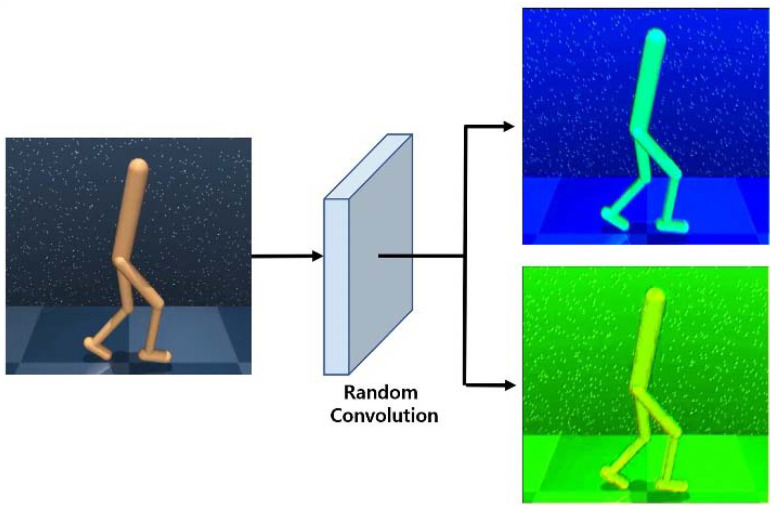
Example of a random convolution process.

When input images pass through a convolutional layer that is randomly initialized in every iteration of reinforcement learning, agents can be trained to be more invariant to the unseen environment. In other words, augmented images, as shown in Figure 4, can significantly improve the generalization of reinforcement learning as they vary the visual patterns of the input data and provide various perturbed low-level features, such as the color, shape, or texture [30]. Although strong data augmentation, such as random convolution, can improve the auxiliary effect on generalization, it cannot be applied independently because it significantly changes the distribution of images, resulting in instability and performance degradation of reinforcement learning.

## 3. Proposed Convolutional–Contrastive Learning for RL (C2RL)

This section describes C2RL, which is a simple, convolutional–contrastive learning architecture that can be attached to reinforcement learning frameworks. First, we describe convolutional–contrastive learning: a novel method to enhance the generalization of vision-based reinforcement learning. Subsequently, we introduce a training method that prevents strong augmentation from degrading the performance of reinforcement learning and maximizes the improvement in the generalization performance in unseen test environments.

### 3.1. Randomized Input Observation

The agent is trained using randomized input observations. To randomize the input observation, a single-layer convolutional neural network is added to the front of the feature extractor as a random network. In each iteration, the parameters of the random network are reinitialized along the Xavier normal distribution [31]. Through the use of the random network, the output has the same dimensions as the input, and various observations with different patterns are generated.

#### Image Blending

**Figure 5:**
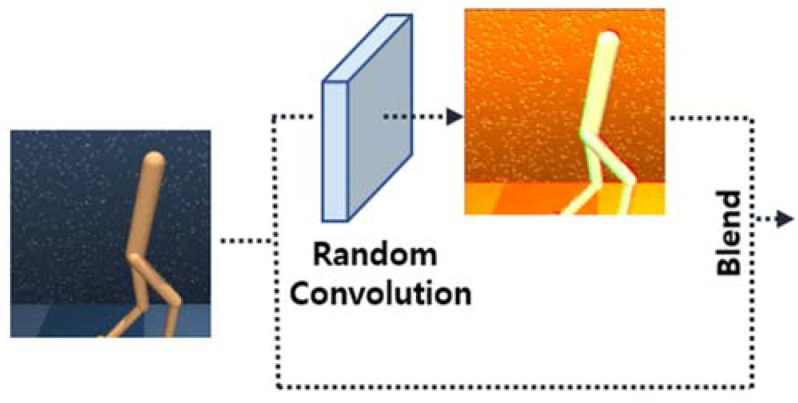
Principle of blending original and randomized images.

To prevent the loss of visual information due to excessive changes in the input image, we blend the image that passes through the random convolutional layer and the original image in a certain proportion, as shown in Figure 5. The image blending ratio is set through parameter α.

(6) $$obs=\alpha \times obs_{origin}+\left(1-\alpha \right)\times obs_{conv}\;\ldots \;\left(0\leq \alpha \leq 1\right)$$

### 3.2. Strong Convolutional–Contrastive Learning

Equation (6) indicates that as α increases, the blending ratio of the original image increases, and convolutional–contrastive learning cannot achieve a sufficient auxiliary effect for the generalization performance. In contrast, when α is small, the large change in the input may confuse reinforcement learning. We introduce a learning method to overcome the trade-off associated with data augmentation strength and effectively exploit strong data augmentation. The training process is divided into two phases, as described in the following subsections.

#### 3.2.1. Self-Pretraining for Strong Augmentation

**Figure 6:**
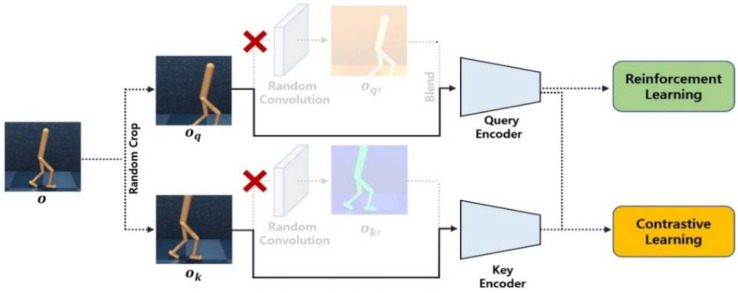
Reinforcement learning and contrastive learning without the random convolution.

In the initial stage of training, random convolution is not applied to the input image. Similar to CURL [12], the query and key representation vectors generated through the encoders are used for reinforcement learning and contrastive learning. As shown in Figure 6, no random convolutional layer is added, and the encoders are trained using only weak data augmentation for contrastive learning. After this self-pretraining process, the agent can use the strongly augmented image more efficiently. Unlike those in normal pretraining, data are self-generated in self-pretraining.

#### 3.2.2. Convolutional–Contrastive Learning Strategy for Reinforcement Learning

**Figure 7:**
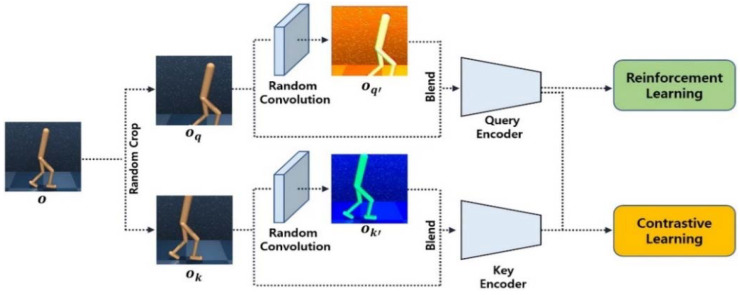
Reinforcement learning and contrastive learning with the random convolution.

After self-pretraining in the early steps of training, a single, random, convolution layer is added to the front of the encoder to induce strong data augmentation as shown in Figure 7. Although strong augmentation is used only during the remaining time, the proposed approach outperforms the training methods that consistently use the same strong augmentation in all stages of training.

## 4. Results

The objective of the proposed approach is to maximize the generalization effect through strong convolution–contrastive learning by preventing the performance degradation of reinforcement learning, owing to the strong augmentation. To evaluate the generalization performance, we compare the scores in various unseen test environments after training the agent via 500 k steps in DMControl [17]. Following the settings of PAD [32], we measure the generalization performance in the two types of test environments, i.e., those involving statically changing background (color-hard mode) and dynamically changing background (video-easy mode). We compare the test scores for the proposed augmentation methods of convolutional–contrastive learning and existing generalization methods. The test score is the average of episode returns obtained using 10 random seeds for each environment. Self-pretraining is performed for 200 k of the 500 k training steps.

### 4.1. Augmentation Methods for Convolutional–Contrastive Learning

**Figure 8:**
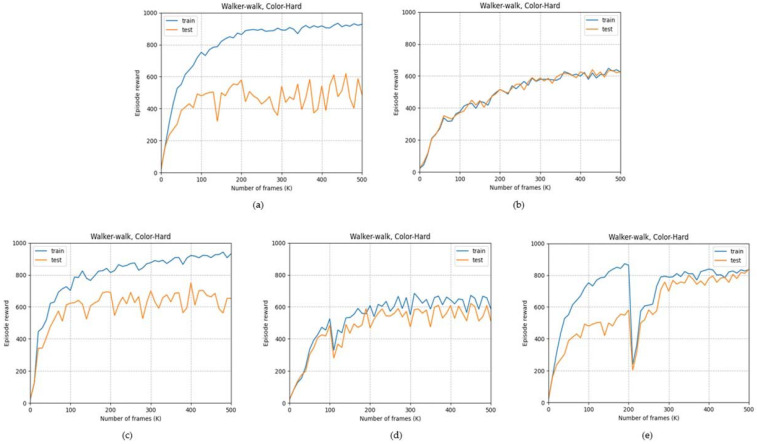
Learning curves on convolutional–contrastive learning. (**a**) uses only original image and (**b**) uses only random image. (**c**,**d**) use blended image with blending parameter α is 0.8 and 0.2 respectively. (**e**) uses blended image with blending parameter α (0.2) after self-pretraining.

We study the effect of various image blending parameters of our method(C2RL) on the generalization performance. Figure 8 shows the test scores for the color-hard mode of DMControl walker–walk environment. As shown in Figure 8a–d, a larger blending ratio of images passing through the random network corresponds to a smaller difference between the training score and test score, albeit with lower scores. In contrast, as shown in Figure 8e, the self-pretraining method proposed in Section 3.2 can help achieve higher scores in the test environment, even with considerable blending of the random images. Although the training and test scores are temporarily reduced when strong augmentation is applied after self-pretraining without random convolution, the proposed approach outperforms other methods that use the same augmentation throughout the training process.

**Figure 9:**
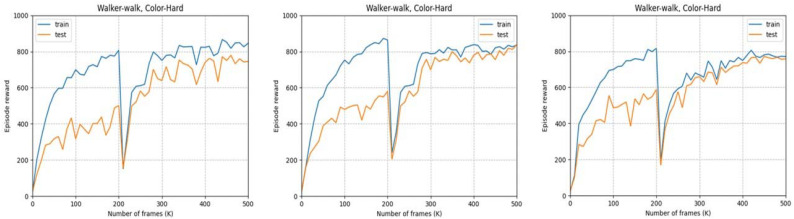
Results for the change in the multiple image blending ratio α after self-pretraining. From the left, 0.5, 0.2 and 0 are used as blending parameters *α*, respectively.

Figure 9 shows the results according to the image blending ratio. After self-pretraining, we compare the results by setting the blending ratio α to 0.5, 0.2, and 0, and also shows the best performance at 0.2. If the blending ratio is 0.5, the generalization effect by random convolution is only half-used. However, we find that when the blending ratio is zero, a large change of the image makes reinforcement learning more difficult.

Moreover, we compare the test scores associated with different blending parameters of C2RL in various unseen environments of DMControl: normal **SAC**; **CURL**: using only weak augmentation(random crop) without random convolution, same as C2RL with α = 1; **C2RL(0.8)**: using a small ratio of random blending (α = 0.8) without self-pretraining; **C2RL(0.2)**: using a large ratio of random blending (α = 0.2) without self-pretraining; and **C2RL(+SP):** C2RL(0.2) with self-pretraining. As shown in Table 1 and Table 2, the highest score is obtained when self-pretraining is used in both modes of DMControl. In other words, self-pretraining allows strong data augmentation to be used efficiently for reinforcement learning and contrastive learning.

### 4.2. Comparison with Existing Reinforcement Learning Networks

We compare the proposed approach with state-of-the-art methods of vision-based reinforcement learning; **CURL [12]**: a contrastive learning method using only weak augm entation (random crop) for reinforcement learning, same as C2RL with α = 1; **RAD [33]**: introduces two new data augmentations, i.e., random translate and random amplitude scale; **DrQ [34]**: uses value function regularization through data augmentation; **PAD [32]**: a self-supervised learning method for policy adaptation during the test. As shown in Table 3 and Table 4, in all environments of DMControl, the proposed method outperforms the state-of-the-art methods.

## 5. Conclusions

This paper proposes a novel, self-supervised learning method named C2RL, which allows the agent to use strong augmented images as the input. Self-pretraining without strong augmentation allows the agents to be trained by efficiently using strong data augmentation. Experimental results on the DMControl suite show that using part of the training process for self-pretraining, without strong augmentation, can promote the more efficient use of strong data augmentation, such as random convolution compared with that when the same strong data augmentation is used throughout the training. Moreover, the proposed method outperforms the state-of-the-art methods in extracting robust visual representations.

## Figures and Tables

**Table 1:** Test scores for different augmentation methods in the DMControl color-hard mode.

Color-Hard	SAC	CURL	C2RL(0.8)	C2RL(0.2)	C2RL(+SP)
Walker, walk	414 ± 74	445 ± 99	707 ± 43	617 ± 46	**899** ± **15**
Walker, stand	719 ± 74	662 ± 54	874 ± 46	912 ± 27	**954** ± **16**
Cartpole, swingup	592 ± 50	454 ± 110	790 ± 59	375 ± 39	**794** ± **20**
Cartpole, balance	857 ± 60	782 ± 13	921 ± 15	970 ± 22	**978** ± **12**
Ball in cup, catch	411 ± 183	231 ± 92	713 ± 166	713 ± 93	**893** ± **44**
Finger, turn_easy	270 ± 43	202 ± 32	438 ± 95	454 ± 133	**464** ± **111**
Cheetah, run	154 ± 41	202 ± 22	251 ± 33	274 ± 13	**292** ± **5**
Reacher, easy	163 ± 45	325 ± 32	317 ± 67	212 ± 91	**332** ± **61**

**Table 2:** Test scores for different augmentation methods in the DMControl video-easy mode.

Video-Easy	SAC	CURL	C2RL(0.8)	C2RL(0.2)	C2RL(+SP)
Walker, walk	616 ± 80	556 ± 133	784 ± 34	689 ± 46	**948** ± **15**
Walker, stand	899 ± 53	852 ± 75	766 ± 47	891 ± 35	**969** ± **23**
Cartpole, swingup	375 ± 90	404 ± 67	589 ± 44	415 ± 38	**600** ± **16**
Cartpole, balance	693 ± 109	850 ± 91	926 ± 13	942 ± 18	**948** ± **12**
Ball in cup, catch	393 ± 175	316 ± 119	692 ± 85	643 ± 93	**747** ± **79**
Finger, turn_easy	355 ± 108	248 ± 56	**461** ± **188**	367 ± 154	421 ± 143
Cheetah, run	194 ± 30	154 ± 50	**287** ± **21**	234 ± 32	265 ± 24

**Table 3:** Learning curves for various augmentation strategies (Color-hard).

Color-Hard	CURL	RAD	DrQ	PAD	C2RL + SP (Ours)
Walker, walk	445 ± 99	400 ± 61	520 ± 91	468 ± 47	**899** ± **15**
Walker, stand	662 ± 54	644 ± 88	770 ± 71	797 ± 46	**954** ± **16**
Cartpole, swingup	454 ± 110	590 ± 53	586 ± 52	630 ± 63	**794** ± **20**
Ball in cup, catch	231 ± 92	541 ± 29	365 ± 210	563 ± 50	**893** ± **44**

**Table 4:** Learning curves for various augmentation strategies (Video-easy).

Video-Easy	CURL	RAD	DrQ	PAD	C2RL + SP (Ours)
Walker, walk	556 ± 133	606 ± 63	682 ± 89	717 ± 79	**948** ± **15**
Walker, stand	852 ± 75	745 ± 146	873 ± 83	935 ± 20	**969 ± 23**
Cartpole, swingup	404 ± 67	373 ± 72	485 ± 105	521 ± 76	**600** ± **16**
Ball in cup, catch	316 ± 119	481 ± 26	318 ± 157	436 ± 55	**747** ± **19**

## Data Availability

Not applicable.
